# The value of combining the simple anthropometric obesity parameters, Body Mass Index (BMI) and a Body Shape Index (ABSI), to assess the risk of non-alcoholic fatty liver disease

**DOI:** 10.1186/s12944-022-01717-8

**Published:** 2022-10-20

**Authors:** Maobin Kuang, Guotai Sheng, Chong Hu, Song Lu, Nan Peng, Yang Zou

**Affiliations:** 1 Medical College of Nanchang University, Nanchang of Jiangxi, 330006 Nanchang, China; 2 Department of Cardiology, Jiangxi Provincial People’s Hospital, 330006 Nanchang, Jiangxi China; 3 Department of Gastroenterology, Jiangxi Provincial People’s Hospital, 330006 Nanchang, Jiangxi China; 4 Jiangxi Cardiovascular Research Institute, Jiangxi Provincial People’s Hospital, 330006 Nanchang, Jiangxi China

**Keywords:** Body mass index, Waist circumference, ABSI, ARI, Receiver operating characteristic analysis, Non-alcoholic fatty liver disease

## Abstract

**Background:**

Body mass index (BMI) and A Body Shape Index (ABSI) are current independent risk factors for non-alcoholic fatty liver disease (NAFLD). The aim of this study was to explore the value of combining these two most common obesity indexes in identifying NAFLD.

**Methods:**

The subjects in this study were 14,251 individuals from the NAfld in the Gifu Area, Longitudinal Analysis (NAGALA) cohort who underwent routine health examination. We integrated BMI with WC and with ABSI to construct 6 combined obesity indicators—obesity phenotypes, the combined anthropometric risk index (ARI) for BMI and ABSI, optimal proportional combination O_BMI+WC_ and O_BMI+ABSI_, and multiplicative combination BMI*WC and BMI*ABSI. Several multivariable logistic regression models were established to evaluate the relationship between BMI, WC, ABSI, and the above six combined indicators and NAFLD; receiver operating characteristic (ROC) curves were drawn to compare the ability of each obesity indicator to identify NAFLD.

**Results:**

A total of 2,507 (17.59%) subjects were diagnosed with NAFLD. BMI, WC, ABSI, and all other combined obesity indicators were significantly and positively associated with NAFLD in the current study, with BMI*WC having the strongest correlation with NAFLD in female subjects (OR per SD increase: 3.13) and BMI*ABSI having the strongest correlation in male subjects (OR per SD increase: 2.97). ROC analysis showed that ARI and O_BMI+ABSI_ had the best diagnostic performance in both sexes, followed by BMI*WC (area under the curve: female 0.8912; male 0.8270). After further age stratification, it was found that ARI and multiplicative indicators (BMI*WC, BMI*ABSI) and optimal proportional combination indicators (O_BMI+WC_, O_BMI+ABSI_) significantly improved the NAFLD risk identification ability of the basic anthropometric parameters in middle-aged females and young and middle-aged males.

**Conclusion:**

In the general population, BMI combined with ABSI best identified obesity-related NAFLD risk and was significantly better than BMI or WC, or ABSI. We find that ARI and the multiplicative combined indicators BMI*WC and BMI*ABSI further improved risk prediction and may be proposed for possible use in clinical practice.

**Supplementary Information:**

The online version contains supplementary material available at 10.1186/s12944-022-01717-8.

## Background

Non-alcoholic fatty liver disease (NAFLD) is a chronic non-infectious liver disease characterized by oxidative stress, inflammation, and fibrosis in hepatocytes among people without a history of excessive alcohol consumption [1, 2]. NAFLD can present as asymptomatic simple hepatic steatosis in its early stages, and without intervention, oxidative stress, inflammation, and fibrosis in hepatocytes can contribute to the continuous progression of NAFLD to non-alcoholic steatohepatitis and then to cirrhosis and ultimately to hepatocellular carcinoma [2, 3]. Recent epidemiological surveys have shown that about a quarter of the global population suffers from NAFLD, with a 27.4% estimated prevalence in Asia [4]. NAFLD is not only the most common chronic liver disease worldwide but has replaced hepatitis B virus infection as the primary risk factor for advanced liver diseases such as cirrhosis and hepatocellular carcinoma [5]. The high prevalence of NAFLD and its severe complications place a heavy burden on humans and the world’s health care systems, making the early prevention, identification, and intervention of NAFLD of great public health importance.

Obesity is one of the most important risk factors for NAFLD [6]. Waist circumference (WC) and body mass index (BMI) are currently the most widely used basic body measures for the assessment of general and central obesity and also are known risk factors for NAFLD [7–10]. Both BMI and WC alone, however, have some distinct limitations, such as the inability of BMI alone to distinguish between adipose and muscle tissue and the inability of WC to determine the differential contribution of abdominal visceral adipose tissue and abdominal subcutaneous adipose tissue to central obesity [11–13]. It is not clear if combining risk associated with BMI and WC can improve the individual risk association. Several relevant studies have analyzed the performance of the combination of BMI and WC in disease risk assessment, and overall, combining BMI and WC in different ways significantly improved the predictive efficacy for obesity-related diseases such as obesity-related hypertension, type 2 diabetes, cardiovascular disease, and all-cause mortality risk compared to a single indicator [14–17]. In addition, a recent study by Wang et al. also found that high levels of WC and BMI were both associated with NAFLD risk in people with type 2 diabetes and that BMI combined with WC was superior to the single measures in assessing the risk of NAFLD [18]. What remains unclear, however, is whether the combination of BMI and WC best identifies a higher risk of NAFLD in the general population. Furthermore, it is important to note that there is a strong correlation between BMI and WC (r ≈ 0.80), and directly combining BMI with WC has the potential to confound the association between BMI and NAFLD, leading to biased and possibly misleading risk estimates [19–21]. A Body Shape Index (ABSI), an allometric abdominal obesity index calculated from WC, height, and weight, is intended to be independent of BMI and has proven to be useful for identifying individuals at risk for sarcopenic obesity [22]; therefore, we also evaluated combining BMI with the non-correlated abdominal obesity index ABSI. This study sought to explore risk assessment for NAFLD of various combinations of BMI and WC or ABSI in a cohort of general health examinees from NAGALA.

## Methods

### Data sources and study design

**Fig. 1:**
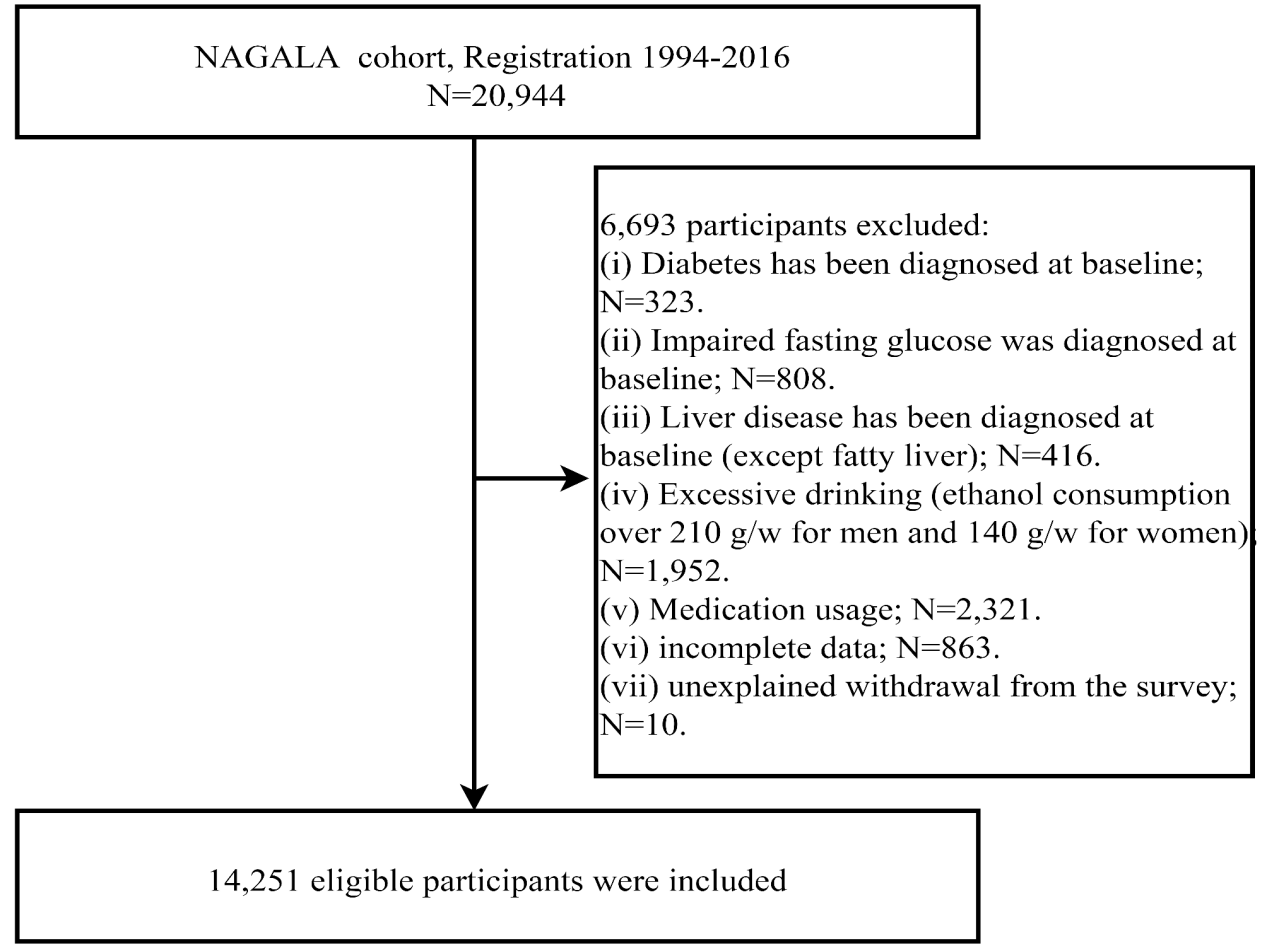
Flowchart of the selection process of study subjects

In the current study, we used data from 14,251 medical examiners recruited by the NAGALA cohort between 1994 and 2016 [23]. NAGALA is an ongoing longitudinal cohort study initiated by Murakami Memorial Hospital in 1994, using physical examination data from the general population to assess common risk factors for the development of chronic diseases; a detailed description of the NAGALA cohort study design has been previously published and the study data have been uploaded by Professor Okamura to the Dryad database for public access [24]. For the current study, we excluded from the original data set (1) subjects who were diagnosed or self-reported with hepatitis (viral/alcoholic) or diabetes and impaired fasting glucose at baseline (n = 1,547); (2) subjects with alcohol abuse (n = 1,952, males consuming ≥ 210 g of alcohol per week and females consuming ≥ 140 g) [25]; (3) subjects taking any medications at baseline (n = 2,321); and, (4) subjects with missing anthropometric and medical examination data (n = 873) were also excluded. We ultimately included 14,251 eligible subjects, with the inclusion and exclusion criteria shown in Fig. 1. The NAGALA cohort study has been ethically reviewed by the Murakami Memorial Hospital Institutional Ethics Review Committee (IRB2018-09-01) and all subjects signed written informed consent for the use of their data; The current study was a post-hoc analysis of the NAGALA cohort, and the Jiangxi Provincial People’s Hospital Ethics Review Committee reviewed the study design and granted approval (IRB2021-066).

In this study, we extracted the following data from each subject: anthropometric and demographic information including weight, height, sex, age, WC, BMI, ABSI, smoking and drinking status, and exercise habits of the subjects; where drinking status was classified according to the subject’s weekly alcohol consumption in the previous month as moderate drinking, light drinking and never or small drinking; smoking status was defined as never, previous and current smoking; for exercise habits, subjects were considered to have exercise habits if they had at least one physical activity of any form per week. BMI was calculated as weight (kg)/[height (m)]^2^ and ABSI was calculated as 1,000*WC (m)*[weight (kg)] ^– 2/3^*[height (m)]^5/6^ [22].

Data on laboratory tests, including blood pressure, triglycerides (TG), aspartate aminotransferase (AST), total cholesterol (TC), fasting blood glucose (FPG), gamma-glutamyl transferase (GGT), high-density lipoprotein cholesterol (HDL-C), alanine aminotransferase (ALT), and glycated hemoglobin (HbA1c). The specific steps for obtaining demographic and anthropometric information and data from laboratory tests have been described in detail elsewhere [19]; all procedures in this study fully followed the Declaration of Helsinki. See STROBE statement (S1 Text).

### Definition and derivation of the combined indicators of BMI with WC and ABSI

Based on previous studies, the present study included four combination types of BMI with WC and ABSI: (1) Using the WHO-recommended clinical cut-offs of WC and BMI combined into four obesity phenotypes; (2) constructing the optimal proportional combination of BMI with WC and ABSI, O_BMI+WC_ and O_BMI+ABSI_; and (3) using the multiplicative combination of WC and ABSI with BMI, BMI*WC, and BMI*ABSI; (4) construction of anthropometric risk index [ARI (BMI, ABSI)] for combined risk of BMI and ABSI using the method recommended by Krakauer NY et al. [26].

### Combination using usual cut-offs for BMI and WC

In accordance with the World Health Organization (WHO) expert committee cut-off recommendations for Asian populations, we defined BMI ≥ 25 kg/m^2^ as overweight/obese and BMI < 25 kg/m^2^ as normal weight [27]. We defined WC ≥ 85 cm for males or WC ≥ 80 cm for females as central obesity and WC < 85 cm for males or WC < 80 cm for females as normal WC [28]. All subjects were assigned to the following four obesity phenotypes according to the baseline WC and BMI: (1) BMI^N^/WC^N^: normal weight + normal WC; (2) BMI^O^/WC^N^: overweight/obese + normal WC; (3) BMI^N^/WC^O^: normal weight + central obesity; (4) BMI^O^/WC^O^: overweight/obese + central obesity.

### Optimal proportional combinations O_**BMI+WC**_ and O_BMI+ABSI_, multiplicative combination indicators BMI*ABSI and BMI*WC, and ARI

The optimal proportional combination indicator O_BMI+WC_ was calculated as O_BMI+WC_= n_WC_*WC + n_BMI_*BMI. The optimal proportion coefficients for WC (n_WC_) and BMI (n_BMI_) were calculated as n_WC_ = β_WC_ / (β_BMI_ + β_WC_) and n_BMI_ = β_BMI_ / (β_BMI_ + β_WC_), respectively; where β_WC_ and β_BMI_ were the regression coefficients of WC and BMI in the multivariable logistic regression model, respectively. To acquire regression coefficients for WC and BMI, we included the presence or absence of NAFLD as the outcome event of interest, WC and BMI as variables in a multivariate-adjusted logistic regression model, and age, height, ALT, AST, systolic blood pressure (SBP), TG, GGT, HDL-C, HbA1c, TC, FPG, and drinking status were adjusted as covariates. Similarly, we used the same method to obtain O_BMI+ABSI_. Since NAFLD risk was positively correlated with BMI, WC, and ABSI, and there was no correlation between BMI and ABSI, which is consistent with the construction of an ARI (BMI, ABSI) for the combined risk of BMI and ABSI, and we calculated ARI (BMI, ABSI) using the method provided by Krakauer NY et al. [26]. In addition, we calculated the products BMI*WC and BMI* ABSI for further analysis.

### Diagnosis of NAFLD

Specialized gastroenterologists, blinded to the subjects scored each abdominal ultrasound for NAFLD risk based on four criteria: (1) changes in the intensity of liver blood flow signals (0–4 points); (2) the clarity of liver blood vessels (0–1 points); (3) liver and kidney echo contrast (0–4 points); (4) deep liver echo attenuation (0–2 points). NAFLD was diagnosed if the sum of the above four scores was greater than 2 [29].

### Statistical analysis

We performed all statistical analysis steps in the current study using Empower (R) version 2.2 and R language version 3.4.3. Significance was defined as *P* < 0.05 in all comparisons (two-sided). Given the significant differences in body composition, fat deposition patterns, and prevalence of NAFLD between males and females [30], all analyses in the present study were performed separately for female and male subjects and the specific steps were as follows:

First, we calculated the quartile categories of BMI*ABSI using the quartile function and subsequently described baseline information by grouping all subjects based on the quartile categories of BMI*ABSI and whether they had NAFLD. For the measurement data, QQ plots were first used to determine the pattern of data distribution, and the data with a normal and a skewed distribution were described as mean (standard deviation) and median (interquartile range), respectively. T-test or one-way ANOVA was used for group comparisons of normal data, and Mann-Whitney rank-sum test or Kruskal-Wallis rank-sum test was used for group comparisons of skewed data. Count data were described as frequencies (%), using the Pearson chi-square test for comparison between groups.

Second, before exploring the association of each obesity indicator with NAFLD, we first analyzed the correlations between several anthropometric measures (height, BMI, WC, ABSI), and it is worth mentioning that a combination of two basic anthropometric indicators of obesity which have smaller correlations is considered more appropriate. Additionally, we also performed a collinearity screening for all covariates [31], where the weight and diastolic blood pressure (DBP) had variance inflation factors (VIF) greater than 5 and were therefore treated as collinear variables and not included in the subsequent model adjustment (Supplementary Table 1). We developed one univariate and three multivariable logistic regression models for assessing the associations between the continuous variables BMI, WC, ABSI, ARI, O_BMI+WC_, O_BMI+ABSI_, BMI*ABSI, BMI*WC, and the categorical variable obesity phenotypes and risk of NAFLD, and recorded the corresponding Odds ratio (OR) and 95% confidence interval (CI), respectively. Due to the large numerical span of BMI*WC (689.56-6490.01) and BMI*ABSI (961.13-3630.94), the change in effect value caused by the change of one unit was small, so the BMI*WC and BMI*ABSI were Z-score transformed and included in the regression models. In the model adjustment of multivariable logistic regression, model 1 considered the effect of anthropometric parameters of age and height on NAFLD; model 2 adjusted drinking status and important lipid parameters (TC, TG, HDL-C) on the basis of model 1; model 3 further considered the effects of liver enzyme indicators (ALT, AST, GGT), blood glucose and blood pressure parameters (FPG, HbA1c, SBP) on the basis of model 2. In addition, to further explore the correlation between BMI*ABSI and NAFLD, we also included BMI*ABSI as a categorical variable in the logistic regression models and calculated the trend of the association between the median of each category of BMI*ABSI and NAFLD.

Third, based on the results of the correlation analysis between each obesity indicator and NAFLD, we also constructed receiver operating characteristic (ROC) curves to evaluate the ability of continuous obesity indicator BMI, WC, ABSI, ARI, O_BMI+WC_, O_BMI+ABSI_, BMI*ABSI, and BMI*WC to identify the risk of NAFLD. The area under the curve (AUC) and Delong test were used to compare the differences in the ability to identify NAFLD risk of each obesity indicator in both sexes and different age subgroups of both sexes, and the optimal diagnostic threshold for each indicator was calculated using Youden’s index.

## Results

### Demographic and clinical characteristics of subjects

The study included 14,251 subjects, of whom 7,411 (52%) were males, with a mean age of 43.82 (8.99) years, and 2,029 (27.37%) were diagnosed with NAFLD; 6,840 were (48%) females, with mean age 43.22 (8.78) years, and 478 (6.99%) were diagnosed with NAFLD. Table 1 is stratified by gender and describes the differences in baseline characteristics between NAFLD patients and non-NAFLD subjects. We found that NAFLD patients consistently had higher age, weight, TC, SBP, DBP, HbA1c, BMI, WC, ABSI, ARI, O_BMI+WC_, O_BMI+ABSI_, BMI*ABSI, BMI*WC, ALT, AST, GGT, FPG, and TG levels in both sexes, but with lower HDL-C levels. Notably, non-NAFLD subjects of both sexes tended to have greater alcohol consumption, which paradoxically appeared to be a protective factor for NAFLD. Additionally, the comparison between the NAFLD group and the non-NAFLD group showed that physical exercise habits were only significantly different in males, height was only significantly different in females, and smoking habits were not significantly different in both sexes.

**Table 1:** Characteristics of 14,251 subjects stratified by NAFLD and gender

	Male				Female		
Variables	Non-NAFLD n = 5382 (72.61%)	NAFLD n = 2029 (27.39%)		*P*-value	Non-NAFLD n = 6362 (93.01%)	NAFLD n = 478 (6.99%)	*P*-value
Age, years	43.7 (9.3)	44.1 (8.2)	0.002		42.9 (8.7)	47.6 (8.3)	< 0.001
Height, cm	170.9 (6.0)	170.6 (5.9)	0.084		158.4 (5.4)	157.0 (5.3)	< 0.001
Weight, kg	64.6 (8.3)	74.3 (10.6)	< 0.001		51.9 (7.1)	63.2 (10.0)	< 0.001
TC, mmol/L	5.1 (0.8)	5.4 (0.9)	< 0.001		5.1 (0.9)	5.6 (0.9)	< 0.001
HDL-C, mmol/L	1.3 (0.3)	1.1 (0.3)	< 0.001		1.7 (0.4)	1.4 (0.3)	< 0.001
SBP, mmHg	116.0 (13.2)	124.0 (14.5)	< 0.001		108.4 (13.8)	120.7 (16.0)	< 0.001
FPG, mmol/L	5.3 (0.4)	5.4 (0.3)	< 0.001		5.0 (0.4)	5.3 (0.4)	< 0.001
DBP, mmHg	72.9 (9.3)	78.4 (10.1)	< 0.001		67.0 (9.5)	75.1 (10.2)	< 0.001
HbA1c, %	5.1 (0.3)	5.3 (0.3)	< 0.001		5.2 (0.3)	5.4 (0.3)	< 0.001
BMI, kg/m2	22.1 (2.4)	25.5 (3.0)	< 0.001		20.7 (2.6)	25.6 (3.6)	< 0.001
WC, cm	78.0 (6.8)	86.6 (7.4)	< 0.001		70.8 (7.3)	83.3 (8.9)	< 0.001
ABSI	75.8 (3.3)	76.7 (3.1)	< 0.001		74.8 (4.6)	76.8 (4.5)	< 0.001
ARI	13.1 (0.9)	14.3 (1.0)	< 0.001		12.2 (1.0)	14.0 (1.3)	< 0.001
O_BMI+WC_	34.4 (3.2)	38.9 (3.8)	< 0.001		31.7 (3.4)	38.3 (4.5)	< 0.001
O_BMI+ABSI_	32.0 (2.1)	34.9 (2.4)	< 0.001		28.9 (2.3)	33.4 (3.0)	< 0.001
BMI*WC	1714.2 (1507.7-1939.1)	2152.9 (1919.2-2457.9)	< 0.001		1423.8 (1249.1-1645.8)	2070.2 (1795.3-2396.7)	< 0.001
BMI*ABSI	1669.3 (1538.1–1806.0)	1922.5 (1795.9-2082.3)	< 0.001		1518.4 (1390.8-1673.5)	1917.8 (1756.7-2131.4)	< 0.001
ALT, U/L	18.0 (14.0–23.0)	29.0 (22.0–41.0)	< 0.001		13.0 (11.0–17.0)	19.0 (15.0–26.0)	< 0.001
AST, U/L	17.0 (14.0–21.0)	21.0 (17.0–26.0)	< 0.001		16.0 (13.0–19.0)	18.0 (15.0–22.0)	< 0.001
GGT, U/L	17.0 (14.0–24.0)	24.0 (18.0–35.0)	< 0.001		12.0 (9.0–14.0)	15.0 (12.0–20.0)	< 0.001
TG, mmol/L	0.8 (0.6–1.2)	1.3 (0.9–1.9)	< 0.001		0.5 (0.4–0.8)	1.0 (0.7–1.4)	< 0.001
Habit of exercise, No. (%)			< 0.001				0.335
No	4300 (79.9%)	1720 (84.8%)			5351 (84.1%)	410 (85.8%)	
Yes	1082 (20.1%)	309 (15.2%)			1011 (15.9%)	68 (14.2%)	
Drinking status, No. (%)			< 0.001				0.004
Non or small	3731 (69.3%)	1623 (80.0%)			5986 (94.1%)	465 (97.3%)	
Light	1096 (20.4%)	273 (13.5%)			376 (5.9%)	13 (2.7%)	
Moderate	555 (10.3%)	133 (6.6%)					
Smoking status, No. (%)			0.067				0.664
Non	1952 (36.3%)	758 (37.4%)			5609 (88.2%)	427 (89.3%)	
Past	1538 (28.6%)	615 (30.3%)			382 (6.0%)	24 (5.0%)	
Current	1892 (35.2%)	656 (32.3%)			371 (5.8%)	27 (5.6%)	

Values were expressed as mean (standard deviation) or medians (quartile interval) or n (%). Abbreviations: BMI: body mass index; WC: waist circumference; ALT: alanine aminotransferase; AST: aspartate aminotransferase; GGT: gamma-glutamyl transferase; HDL-C: high-density lipoprotein cholesterol; TC: total cholesterol; TG: triglyceride; FPG: fasting plasma glucose; HbA1c: hemoglobin A1c; SBP: systolic blood pressure; DBP: diastolic blood pressure; NAFLD: non-alcoholic fatty liver disease; ABSI: A body shape index; O_BMI+ABSI_: Optimal proportional combination of BMI and ABSI; O_BMI+WC_: Optimal proportional combination of BMI and WC; ARI: Anthropometric risk index

Baseline characteristics for all subjects according to the quartile category of BMI*ABSI are shown in Table 2. We found that with the increase of BMI*ABSI quartiles, all baseline metrics of subjects showed a trend of change (all *P* < 0.001). Of these, a greater proportion of male subjects were in the higher BMI*ABSI quartiles, with a male-to-female ratio of almost 7:3 in Q4, and there were more subjects with smoking and drinking habits and with less physical exercise in Q4. Moreover, subjects’ HDL-C levels decreased with increasing BMI*ABSI quartiles, while age, BMI, WC, ABSI, ARI, O_BMI+WC_, O_BMI+ABSI_, BMI*WC, height, weight, TC, SBP, HbA1c, ALT, AST, DBP, FPG, GGT, TG levels, and the prevalence of NAFLD increased (Q4: 48.5%).

**Table 2:** Characteristics of 14,251 subjects stratified according to the BMI*ABSI quartiles

	BMI*ABSI quartiles				
variables	Q1 (961.1-1477.2)	Q2 (1477.4-1645.5)	Q3 (1645.6-1827.4)	Q4 (1827.5-3630.9)	*P*-value
No. of subjects	3563	3562	3563	3563	
Male, No. (%)	867 (24.3%)	1666 (46.8%)	2269 (63.7%)	2609 (73.2%)	
Age, years	41.0 (8.7)	43.1 (8.7)	44.5 (8.6)	45.6 (8.9)	< 0.001
BMI, kg/m^2^	18.7 (1.3)	20.9 (1.2)	22.7 (1.2)	26.0 (2.5)	< 0.001
WC, cm	65.4 (3.7)	72.8 (2.9)	78.8 (2.9)	87.7 (5.8)	< 0.001
ABSI	73.1 (3.8)	75.1 (3.8)	76.5 (3.5)	77.5 (3.5)	< 0.001
ARI	11.4 (0.4)	12.4 (0.3)	13.2 (0.3)	14.4 (0.8)	< 0.001
O_BMI+WC_	29.0 (1.5)	32.3 (1.0)	35.0 (1.1)	39.6 (3.0)	< 0.001
O_BMI+ABSI_	27.4 (1.3)	29.9 (1.1)	31.9 (1.1)	35.0 (2.0)	< 0.001
BMI*WC	1241.2 (1147.1-1318.4)	1514.2 (1447.2-1587.4)	1782.6 (1703.2-1865.8)	2194.4 (2051.7-2436.9)	< 0.001
Height, cm	161.7 (7.7)	164.3 (8.3)	166.1 (8.5)	167.1 (8.4)	< 0.001
Weight, kg	49.0 (5.7)	56.4 (6.5)	62.7 (7.3)	72.9 (10.3)	< 0.001
TC, mmol/L	4.9 (0.8)	5.0 (0.9)	5.2 (0.9)	5.4 (0.9)	< 0.001
HDL-C, mmol/L	1.7 (0.39)	1.5 (0.4)	1.4 (0.4)	1.2 (0.3)	< 0.001
SBP, mmHg	105.6 (12.5)	111.1 (12.8)	115.9 (13.3)	123.1 (14.8)	< 0.001
FPG, mmol/L	4.9 (0.4)	5.1 (0.4)	5.2 (0.4)	5.3 (0.4)	< 0.001
DBP, mmHg	65.5 (8.5)	69.0 (9.1)	72.5 (9.6)	77.6 (10.2)	< 0.001
HbA1c, %	5.1 (0.3)	5.1 (0.3)	5.2 (0.3)	5.3 (0.3)	< 0.001
ALT, U/L	14.0 (11.0–17.0)	15.0 (12.0–19.0)	18.0 (13.0–23.0)	23.0 (17.0–33.0)	< 0.001
AST, U/L	16.0 (13.0–19.0)	16.00 (14.0–20.0)	17.0 (14.0–21.0)	19.0 (16.0–24.0)	< 0.001
GGT, U/L	12.0 (10.0–15.0)	13.00 (11.0–18.0)	16.0 (12.0–22.0)	21.0 (15.0–30.0)	< 0.001
TG, mmol/L	0.51 (0.4–0.7)	0.6 (0.5–0.9)	0.8 (0.6–1.2)	1.1 (0.8–1.6)	< 0.001
Habit of exercise, No. (%)					< 0.001
No	2963 (83.2%)	2858 (80.2%)	2920 (82.0%)	3040 (85.3%)	
Yes	600 (16.8%)	704 (19.8%)	643 (18.1%)	523 (14.7%)	
Drinking status, No. (%)					< 0.001
Non or small	3228 (90.6%)	2937 (82.5%)	2848 (79.9%)	2792 (78.4%)	
Light	279 (7.8%)	467 (13.1%)	507 (14.2%)	505 (14.2%)	
Moderate	56 (1.6%)	158 (4.4%)	208 (5.8%)	266 (7.5%)	
Smoking status, No. (%)					< 0.001
Non	2736 (76.8%)	2322 (65.2%)	1957 (54.9%)	1731 (48.6%)	
Past	337 (9.5%)	560 (15.7%)	785 (22.0%)	877 (24.6%)	
Current	490 (13.8%)	680 (19.1%)	821 (23.0%)	955 (26.8%)	
NAFLD, No. (%)	16 (0.5%)	150 (4.2%)	611 (17.2%)	1730 (48.5%)	< 0.001

Abbreviations as in Table 1

### Calculation of the optimal proportional combination of BMI with WC and ABSI

In the logistic regression equation constructed to calculate the optimal proportional combination O_BMI+WC_, the regression coefficient β_BMI_ for BMI was 0.218 (0.154–0.282), and β_WC_ for WC was 0.063 (0.038–0.089) in females; in males, β_BMI_ was 0.177 (0.124–0.299), and β_WC_ was 0.068 (0.048–0.089). Therefore, according to the calculation formula of the optimal proportional coefficients, the values ​​of n_BMI_ and n_WC_ in females were 0.776 and 0.224, respectively, and the values ​​of n_BMI_ and n_WC_ in males were 0.722 and 0.278, respectively. Similarly, in the logistic regression equation constructed to calculate the optimal proportional combination O_BMI+ABSI_, β_BMI_ and β_ABSI_ were 0.356 and 0.064, respectively, in females, resulting in n_BMI_ and n_ABSI_ values ​​of 0.848 and 0.152, respectively; in males, β_BMI_ and β_ABSI_ were 0.334 and 0.075, respectively, and n_BMI_ and n_ABSI_ values ​​were 0.817 and 0.183, respectively. Table 3 shows the calculation formulas of O_BMI+WC_ and O_BMI+ABSI_.

**Table 3:** Optimal combination equations of BMI with WC and ABSI.

	Logistic regression derived equations	
	Male	Female
equations for BMI and WC	0.722BMI + 0.278WC	0.776BMI + 0.224WC
equations for BMI and ABSI	0.817BMI + 0.183ABSI	0.848BMI + 0.152ABSI

Abbreviations as in Table 1

### Association of various obesity indicators with NAFLD

Correlation analysis of the basic anthropometric measures showed a strong correlation between BMI and WC in both sexes (r: male 0.8796; female 0.8191), while BMI was barely correlated with ABSI (r < 0.05) (Supplementary Table 2). In contrast, the combination of ABSI and BMI may be more appropriate. Table 4 presents the associations between BMI, WC, ABSI, ARI, O_BMI+WC_, O_BMI+ABSI_, BMI*ABSI, and BMI*WC and the risk of NAFLD in both sexes. In multivariate-adjusted logistic regression models, BMI and WC, and ABSI were positively correlated with NAFLD risk. In the current study, the combined indicators ARI, BMI*WC, BMI*ABSI, O_BMI+ABSI_, and O_BMI+WC_ were positively correlated with NAFLD risk in all four logistic regression models, and the OR values ​​in the univariate logistic regression model ranged from 1.44 to 5.02; after further adjustment for age, height, GGT, TC, AST, HDL-C, drinking status, ALT, TG, FPG, HbA1c, and SBP (Model 3), the results did not change significantly. The risk of NAFLD increased by 33% (OR 1.33, 95%CI 1.29, 1.37) and 52% (OR 1.52, 95%CI 1.45, 1.60) and 172% (OR 2.72, 95%CI 2.43, 3.04), in females, for each unit increase in O_BMI+WC_ and O_BMI+ABSI_ and ARI respectively. Similarly, in males, NAFLD risk was increased by 30% (OR 1.30, 95%CI 1.27, 1.33) and 50% (OR 1.50, 95%CI 1.45, 1.56) and 172% (OR 2.72, 95%CI 2.48, 2.98), respectively. Each standard deviation increase in BMI*WC and BMI*ABSI in females was associated with a 213% (OR 3.13, 95%CI 2.74,3,56) and 209% (OR 3.09, 95%CI 2.72, 3.52) increased risk for NAFLD respectively, and for males 185% (OR 2.85, 95%CI 2.59, 3.15) and 197% (OR 2.97, 95%CI 2.68, 3.28), respectively. Furthermore, the association between BMI*ABSI and NAFLD remained unchanged after treating BMI*ABSI as a categorical variable; taking the first quartile as the control group, the NAFLD risk increased with the increase of BMI*ABSI quartiles (all *P* for trend < 0.001). Moreover, we also analyzed the risk of NAFLD for each obesity phenotype in multivariable logistic regression models (Supplementary Table 3), which in model 3 showed that compared with the BMI^N^/WC^N^ phenotype, male and female BMI^O^/ WC^N^, BMI^N^/WC^O^, and BMI^O^/WC^O^ phenotypes all had a significantly higher risk of NAFLD. The risk of NAFLD associated with the BMI^O^/ WC^N^, BMI^N^/WC^O^, and BMI^O^/WC^O^ phenotypes had ORs of 3.67, 3.77, and 9.78 in females and 2.76, 2.74, and 4.19 in males, respectively. Clearly, subjects with the BMI^O^/WC^O^ phenotype consistently had the highest risk of developing NAFLD.

**Table 4:** Odds ratios and 95% confidence intervals of BMI, WC, ABSI, O_BMI+WC_, O_BMI+ABSI_, BMI*ABSI, BMI*WC, and ARI for NAFLD in female and male subjects

	Odds ratios (95% confidence interval)			
	Crude model	Model 1	Model 2	Model 3
Female				
BMI	1.58 (1.53, 1.64)^*^	1.58 (1.52, 1.63)^*^	1.49 (1.44, 1.55)^*^	1.42 (1.36, 1.47)^*^
WC	1.19 (1.17, 1.21)^*^	1.19 (1.17, 1.21)^*^	1.17 (1.15, 1.18)^*^	1.14 (1.12, 1.16)^*^
ABSI	1.09 (1.07, 1.12)^*^	1.07 (1.05, 1.09)^*^	1.06 (1.04, 1.08)^*^	1.05 (1.02, 1.07)^*^
ARI	3.70 (3.36, 4.07)^*^	3.61 (3.28, 3.98)^*^	3.11 (2.80, 3.44)^*^	2.72 (2.43, 3.04)^*^
O_BMI+ABSI_	1.73 (1.66, 1.80)^*^	1.71 (1.65, 1.79)^*^	1.61 (1.54, 1.68)^*^	1.52 (1.45, 1.60)^*^
O_BMI+WC_	1.44 (1.41, 1.48)^*^	1.44 (1.40, 1.48)^*^	1.38 (1.34, 1.42)^*^	1.33 (1.29, 1.37)^*^
BMI*ABSI (Per SD)	4.49 (4.02, 5.02)^*^	4.33 (3.87, 4.84)^*^	3.63 (3.23, 4.09)^*^	3.09 (2.72, 3.52)^*^
BMI*WC (Per SD)	4.50 (4.03, 5.03)^*^	4.41 (3.94, 4.93)^*^	3.68 (3.27, 4.15)^*^	3.13 (2.74, 3.56)^*^
BMI*ABSI (quartiles)				
Quartile 1	1.0	1.0	1.0	1.0
Quartile 2	10.90 (4.64, 25.59)^*^	10.32 (4.39, 24.24)^*^	8.42 (3.57, 19.86)^*^	7.47 (3.16, 17.65)^*^
Quartile 3	43.73 (19.20, 99.63)^*^	39.09 (17.13, 89.20)^*^	25.14 (10.94, 57.76)^*^	19.49 (8.44, 44.98)^*^
Quartile 4	217.88 (96.72, 490.84)^*^	188.51 (83.50, 425.59)^*^	105.60 (46.43, 240.20)^*^	63.97 (27.87, 146.85)^*^
*P* for trend	< 0.0001	< 0.0001	< 0.0001	< 0.0001
Male				
BMI	1.62 (1.58, 1.67)^*^	1.62 (1.58, 1.67)^*^	1.52 (1.48, 1.57)^*^	1.38 (1.34, 1.42)^*^
WC	1.19 (1.18, 1.20)^*^	1.21 (1.20, 1.22)^*^	1.18 (1.17, 1.19)^*^	1.13 (1.12, 1.15)^*^
ABSI	1.09 (1.07, 1.10)^*^	1.10 (1.08, 1.12)^*^	1.06 (1.04, 1.08)^*^	1.05 (1.02, 1.07)^*^
ARI	4.39 (4.06, 4.75)^*^	4.40 (4.07, 4.77)^*^	3.68 (3.38, 4.00)^*^	2.72 (2.48, 2.98)^*^
O_BMI+ABSI_	1.83 (1.77, 1.89)^*^	1.83 (1.77, 1.89)^*^	1.70 (1.65, 1.76)^*^	1.50 (1.45, 1.56)^*^
O_BMI+WC_	1.47 (1.44, 1.50)^*^	1.47 (1.44, 1.50)^*^	1.41 (1.38, 1.44)^*^	1.30 (1.27, 1.33)^*^
BMI*ABSI (Per SD)	5.02 (4.61, 5.48)^*^	5.08 (4.66, 5.55)^*^	4.16 (3.80, 4.56)^*^	2.97 (2.68, 3.28)^*^
BMI*WC (Per SD)	4.72 (4.34, 5.13)^*^	4.83 (4.44, 5.26)^*^	3.95 (3.62, 4.32)^*^	2.85 (2.59, 3.15)^*^
BMI*ABSI (quartiles)				
Quartile 1	1.0	1.0	1.0	1.0
Quartile 2	5.76 (3.00, 11.09)^*^	5.89 (3.06, 11.33)^*^	4.68 (2.42, 9.02)^*^	3.97 (2.03, 7.78)^*^
Quartile 3	23.97 (12.75, 45.07)^*^	25.00 (13.29, 47.03)^*^	15.83 (8.38, 29.90)^*^	10.27 (5.35, 19.72)^*^
Quartile 4	102.03 (54.44, 191.23)^*^	108.71 (57.95, 203.93)^*^	58.44 (30.99, 110.21)^*^	26.15 (13.61, 50.25)^*^
*P* for trend	< 0.0001	< 0.0001	< 0.0001	< 0.0001

^*^P < 0.0001; Abbreviation: SD: standard deviation; other abbreviations as in Table 1

Model 1 adjusted for age and height

Model 2 adjusted for model 1 plus TC, TG, HDL-C, drinking status

Model 3 adjusted for model 2 plus ALT, AST, GGT, FPG, HbA1c, and SBP.

### Accuracy of various obesity indicators to identify NAFLD

**Fig. 2:**
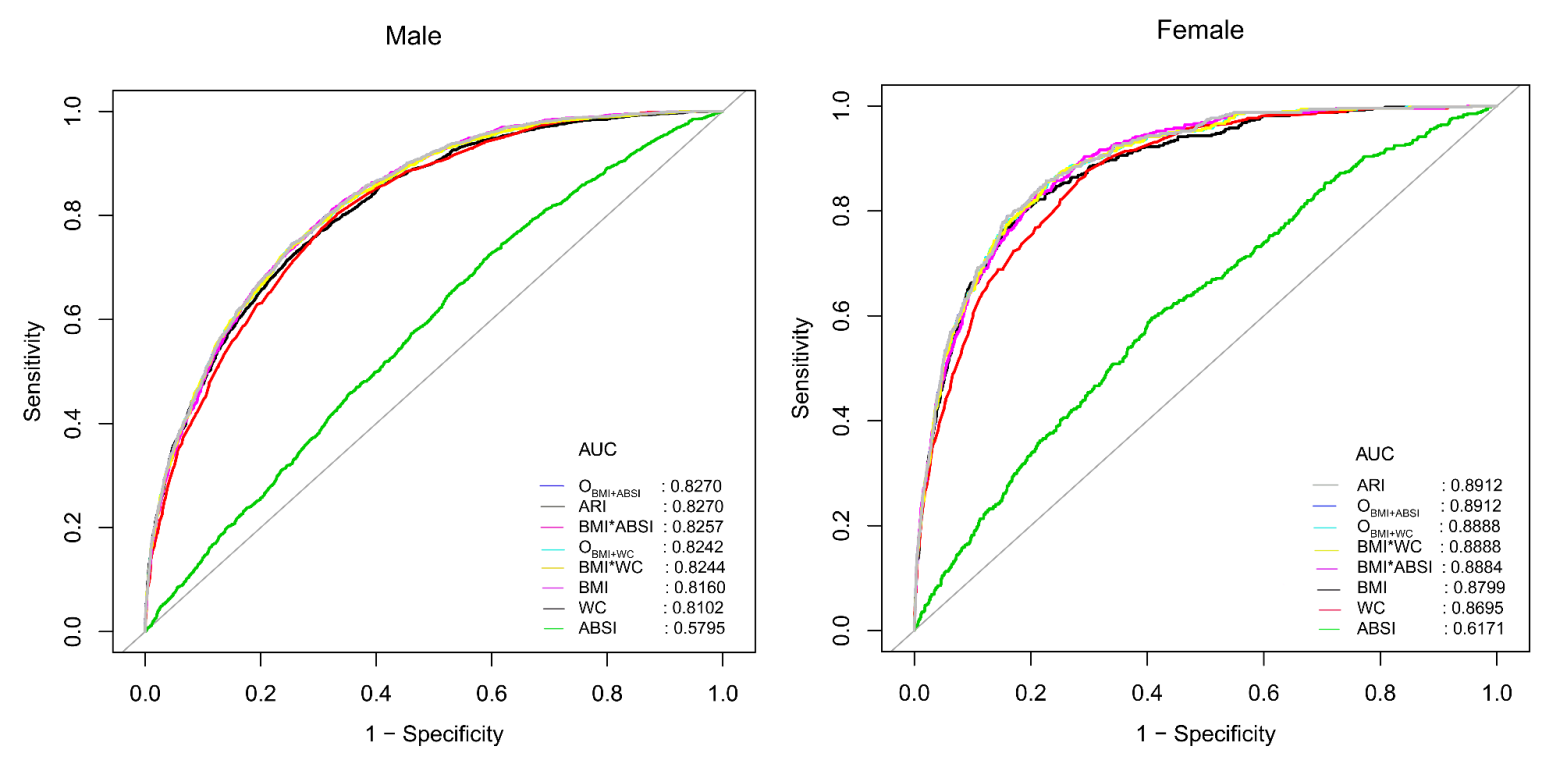
ROC curve analysis of BMI, WC, ABSI, ARI, O_BMI+WC_, O_BMI+ABSI_, BMI*ABSI, and BMI*WC in females and males

ROC curves were drawn to assess the accuracy of BMI, WC, ABSI, ARI, BMI*WC, BMI*ABSI, O_BMI+ABSI_, and O_BMI+WC_ for identifying NAFLD in both sexes (Fig. 2). Table 5 summarizes the AUC and optimal diagnostic thresholds for these obesity indicators. In contrast, ARI and O_BMI+ABSI_ had the same and highest AUC in both female and male subjects, with AUC and optimal diagnostic thresholds of 0.8270, 33.2569 and 0.8912, 30.4143 for ARI in males and females, respectively. The Delong test further showed that both the combined indicators of BMI and WC (O_BMI+WC_ and BMI*WC) and BMI and ABSI (O_BMI+ABSI_ and BMI*ABSI and ARI) were significantly better at identifying the risk of NAFLD than BMI or WC or ABSI alone (Delong test *P* < 0.05).

**Table 5:** The best threshold, sensitivities, specificities, Youden’s index, and area under the curve of BMI*WC, BMI*ABSI, O_BMI+ABSI_, O_BMI+WC_, BMI, WC, ABSI, and ARI in different genders

	AUC	95%CI low	95%CI up	Best threshold	Specificity	Sensitivity	Youden’s index
Female							
BMI^**^	0.8799	0.8648	0.8950	22.7565	0.8177	0.7950	0.6127
WC^**^	0.8695	0.8545	0.8844	74.2500	0.7050	0.8745	0.5804
ABSI^**^ ARI^**^	0.6171 0.8912	0.5916 0.8775	0.6426 0.9048	75.7925 12.7825	0.5926 0.7735	0.5962 0.8577	0.1888 0.6312
O_BMI+ABSI_^**^	0.8912	0.8775	0.9048	30.4143	0.7733	0.8577	0.6310
O_BMI+WC_	0.8888	0.8750	0.9027	34.1234	0.7716	0.8556	0.6272
BMI*ABSI	0.8884	0.8748	0.9021	1704.2100	0.7922	0.8285	0.6207
BMI*WC	0.8888	0.8750	0.9027	1674.1183	0.7733	0.8556	0.6286
Male							
BMI^**^	0.8160	0.8055	0.8264	23.5555	0.7382	0.7309	0.4691
WC^**^	0.8102	0.7998	0.8207	80.6500	0.6674	0.8034	0.4708
ABSI^**^	0.5795	0.5653	0.5936	74.9128	0.4052	0.7240	0.1292
ARI^**^	0.8270	0.8170	0.8369	13.6096	0.7456	0.7457	0.4913
O_BMI+ABSI_^**^	0.8270	0.8170	0.8369	33.2569	0.7458	0.7457	0.4915
O_BMI+WC_	0.8242	0.8141	0.8343	39.8313	0.7438	0.7447	0.4885
BMI*ABSI	0.8257	0.8157	0.8356	1758.7590	0.6761	0.8117	0.4878
BMI*WC	0.8244	0.8143	0.8345	1944.0433	0.7551	0.7329	0.4880

Abbreviations as in Table 1

^**^P < 0.05 compared with BMI*WC in each gender

To explore the changes in BMI, WC, ABSI, ARI, BMI*WC, BMI*ABSI, O_BMI+ABSI_, and O_BMI+WC_ on the risk identification ability of NAFLD in different age subgroups of both sexes, we constructed ROC curves for 20–39, 40–59, and ≥ 60 years in both sexes, respectively; and the corresponding AUC and optimal diagnostic thresholds were summarized in Table 6. We found that ARI and O_BMI+ABSI_ exhibited considerably higher AUCs in all age subgroups for both sexes except in the female ≥ 60-year age group; the AUC of O_BMI+ABSI_ in the female 20–39 age group was 0.9523, the highest value among all age subgroups of both sexes. In the male 20-59-year-old group and the female 40-59-year-old group, the AUC values ​​of ARI and O_BMI+ABSI_ were slightly higher than those of BMI*WC, but all were significantly higher than those of BMI or WC or ABSI alone, while in the female 20–39 years group and the female ≥ 60 years group AUC values for all combined obesity indicators were significantly higher than that of WC and ABSI; in addition, in the male ≥ 60-year-old group, the seven indicators of obesity had similar NAFLD identification abilities, only ABSI underperformed. Therefore, the combined indicators ARI, BMI*WC, BMI*ABSI, O_BMI+ABSI_, and O_BMI+WC_ can significantly improve the ability of simple obesity parameters BMI and WC and ABSI to identify NAFLD in middle-aged females and young and middle-aged males and had the highest diagnostic performance in young females.

**Table 6:** The area under the curve, best threshold, sensitivities, specificities, and Youden’s index of BMI*WC, BMI*ABSI, O_BMI+ABSI_, O_BMI+WC_, BMI, WC, ABSI, and ARI as a predictor of NAFLD in the different age groups of both genders

	AUC	95%CI low		95%CI up		Best threshold	Specificity	Sensitivity	Youden’s index	
Female										
Age 20–39 years										
BMI	0.9511		0.9336		0.9685	23.1266	0.8981	0.8723	0.7704	
WC^***^	0.9319		0.9079		0.9559	76.6000	0.8560	0.8830	0.7390	
ABSI^***^	0.5686		0.5085		0.6288	75.5742	0.6122	0.5319	0.1441	
ARI	0.9522		0.9337		0.9708	12.7922	0.8498	0.9362	0.7860	
BMI*WC	0.9516		0.9331		0.9701	1675.4784	0.8436	0.9255	0.7691	
BMI*ABSI	0.9486		0.9289		0.9682	1701.3486	0.8614	0.9255	0.7869	
O_BMI+ABSI_	0.9523		0.9337		0.9708	30.4368	0.8498	0.9362	0.7860	
O_BMI+WC_	0.9516		0.9330		0.9701	34.1296	0.8424	0.9255	0.7679	
Age 40–59 years										
BMI^***^	0.8484		0.8282		0.8686	22.7527	0.7841	0.7744	0.5585	
WC^***^	0.8423		0.8230		0.8616	74.2500	0.6630	0.8691	0.5321	
ABSI^***^	0.6186		0.5897		0.6476	75.7925	0.5808	0.6072	0.1880	
ARI^***^	0.8622		0.8438		0.8805	13.0283	0.8077	0.7744	0.5821	
BMI*WC	0.8603		0.8417		0.8788	1674.1183	0.7332	0.8384	0.5716	
BMI*ABSI	0.8602		0.8420		0.8785	1704.2100	0.7546	0.8134	0.5680	
O_BMI+ABSI_^***^	0.8622		0.8438		0.8805	30.9995	0.8079	0.7744	0.5823	
O_BMI+WC_	0.8602		0.8417		0.8788	35.0363	0.8093	0.7632	0.5725	
Age ≥ 60 years										
BMI	0.8308		0.7458		0.9159	23.3334	0.8439	0.7600	0.6039	
WC^***^	0.7696		0.6778		0.8613	79.7500	0.7707	0.6800	0.4507	
ABSI^***^	0.5660		0.4551		0.6770	78.1836	0.5024	0.7200	0.2224	
ARI	0.8195		0.7391		0.8999	12.9252	0.7024	0.8400	0.5424	
BMI*WC	0.8199		0.7396		0.9002	1686.1280	0.6976	0.8400	0.5376	
BMI*ABSI	0.8014		0.7186		0.8841	1659.0994	0.5610	0.9600	0.5210	
O_BMI+ABSI_	0.8195		0.7392		0.8998	30.7525	0.7024	0.8400	0.5424	
O_BMI+WC_	0.8201		0.7393		0.9009	34.3393	0.7024	0.8400	0.5424	
Male										
Age 20–39 years										
BMI^***^	0.8439		0.8275		0.8602	23.4782	0.7495	0.7665	0.5160	
WC^***^	0.8415		0.8255		0.8575	80.4500	0.7219	0.8009	0.5228	
ABSI^***^	0.6063		0.5826		0.6300	74.5090	0.4871	0.6991	0.1862	
ARI^***^	0.8549		0.8396		0.8702	13.5026	0.7600	0.7844	0.5444	
BMI*WC	0.8513		0.8357		0.8669	1862.5415	0.7219	0.8114	0.5333	
BMI*ABSI^***^	0.8552		0.8399		0.8704	1766.2886	0.7557	0.7874	0.5431	
O_BMI+ABSI_^***^	0.8549		0.8396		0.8702	33.0076	0.7614	0.7829	0.5443	
O_BMI+WC_	0.8514		0.8358		0.8669	39.1453	0.7290	0.8084	0.5374	
Age 40–59 years										
BMI^***^	0.8007		0.7867		0.8147	23.2505	0.6779	0.7639	0.4418	
WC^***^	0.7908		0.7766		0.8051	81.2500	0.6583	0.7795	0.4378	
ABSI^***^	0.5615		0.5429		0.5801	75.4294	0.3897	0.7005	0.0902	
ARI^***^	0.8104		0.7969		0.8240	13.6142	0.7232	0.7451	0.4683	
BMI*WC	0.8080		0.7943		0.8217	1955.1203	0.7448	0.7177	0.4625	
BMI*ABSI	0.8086		0.7950		0.8222	1816.1016	0.7334	0.7287	0.4621	
O_BMI+ABSI_^***^	0.8105		0.7969		0.8240	33.2733	0.7239	0.7443	0.4682	
O_BMI+WC_	0.8075		0.7938		0.8212	40.1192	0.7475	0.7185	0.4660	
Age ≥ 60 years										
BMI	0.7903		0.7363		0.8444	23.7420	0.8111	0.6951	0.5062	
WC	0.7804		0.7282		0.8326	80.5000	0.5944	0.8659	0.4603	
ABSI^***^	0.5804		0.5119		0.6490	75.8542	0.2941	0.8659	0.1600	
ARI	0.7949		0.7437		0.8462	13.5819	0.6780	0.8171	0.4951	
BMI*WC	0.8029		0.7513		0.8545	1932.6121	0.7368	0.7927	0.5295	
BMI*ABSI	0.7904		0.7393		0.8414	1800.3670	0.6594	0.8293	0.4887	
O_BMI+ABSI_	0.7950		0.7438		0.8462	33.1880	0.6780	0.8171	0.4951	
O_BMI+WC_	0.8023		0.7509		0.8536	39.7476	0.7121	0.8171	0.5292	

Abbreviations as in Table 1

^***^P < 0.05 compared with BMI*WC in each age group of both genders

## Discussion

Our main findings showed that BMI, WC, ABSI, ARI, BMI*WC, BMI*ABSI, O_BMI+ABSI_, O_BMI+WC_, and the obesity phenotypes were all significantly and positively correlated with NAFLD risk; and for the first time in the general population, it was found that BMI combined with WC and ABSI can significantly improve the ability of the basic anthropometric measures to identify NAFLD, especially in middle-aged females and young and middle-aged males. Of significant mention, compared with other age stages in both sexes, all obesity indicators in this study have the highest diagnostic efficiency among young females.

In recent years, the rapid global economic development, dietary patterns and lifestyles of the residents have changed dramatically, and a high-calorie, high-fat daily diet and a sedentary lifestyle have become the main themes of today’s society; therefore, the prevalence of metabolic syndrome, obesity, and other obesity-related diseases has increased greatly [2, 32, 33]. NAFLD is considered the hepatic manifestation of the metabolic syndrome and encompasses a complex spectrum of liver pathologies ranging from simple hepatic steatosis to non-alcoholic steatohepatitis, and also the most common chronic liver disease and the leading cause of liver-related death worldwide [1, 34]. The occurrence and development of NAFLD are closely related to the global epidemic of obesity [6]. Recent epidemiological surveys have shown that the incidence of NAFLD in obese patients can reach 80%, and in severely obese patients who require bariatric surgery, the incidence of NAFLD is as high as 90% [4].

Independent associations between NAFLD and traditional obesity indicators BMI and WC have been demonstrated in many observational studies, but there is much debate as to which indicator is better at identifying obesity-related NAFLD risk [7–10, 35–39]. Several recent studies have demonstrated that BMI was a stronger indicator of obesity for identifying and predicting NAFLD risk, with BMI having the largest AUC compared with other obesity-related parameters, showing better diagnostic performance than WC and other obesity indicators [37–39]. Of course, there were other studies that expressed the opposite standpoints; two studies of NAFLD in obese adolescents and children found that both WC and BMI independently predicted NAFLD risk, with WC, in particular, having the highest predictive accuracy [35, 36]. Furthermore, a meta-analysis study by Qing et al. found that WC remained the strongest anthropometric predictor of NAFLD even after adjusting for a large number of confounding factors, including BMI, and that central obesity was a more dangerous pattern of body fat distribution than general obesity [7]. Clearly, both BMI and WC were independent risk factors for NAFLD, but neither BMI nor WC alone appeared to be an adequate measure of obesity-related NAFLD risk. In a study by Janssen et al. [40], it was found that either BMI or WC alone was an independent predictor of total fat, non-abdominal fat, abdominal subcutaneous fat, and visceral fat, but 20–40% of the variation in fat mass in these fat pools remained unexplained by BMI or WC alone; after they further combining BMI with WC, found that the variability of fat content in these fat pools decreased significantly. Given that the available evidence generally supports both abdominal subcutaneous fat and visceral fat as independent predictors of insulin resistance [41, 42], we hypothesized that the combination of WC and BMI may be a better predictor of metabolic disease. This speculation has been confirmed in studies of metabolic diseases such as cardiovascular disease, obesity-related hypertension, type 2 diabetes, and risk of all-cause mortality [14–17]. Furthermore, in a recent study, Wang et al. also found that BMI combined with WC was more strongly associated with NAFLD in a diabetic population than either BMI or WC alone [18]. However, their study subjects were limited to the diabetic population and did not explore the value of combined BMI and WC in the identification of NAFLD. In addition, it is also worth noting that in the above-mentioned studies related to BMI combined with WC, almost all researchers ignored the statistical artifacts that may result from the strong correlation between BMI and WC. A recent study by Christakoudi S et al. found a traditional U-shaped association between BMI alone and all-cause mortality risk, but when BMI was combined with WC, which had a strong correlation, a predominantly negative association was found between BMI and all-cause mortality, i.e., the higher the BMI the lower the mortality risk [19]. It means that the combination of WC and BMI may not simply supplement BMI, but even changed the pattern of associations between BMI and outcome events, which may be a statistical artifact caused by the strong correlation between WC and BMI. In addition, when they combined BMI with the moderately correlated waist index waist-to-height ratio found a similar but more modest change in the pattern of association between BMI and all-cause mortality. Therefore, combining BMI with a correlated abdominal obesity index may lead to biased and possibly even misleading risk estimates and ineffective risk stratification [19]. As expected, when the researchers further combined BMI with the unrelated abdominal obesity index ABSI and hip circumference, they found that the pattern of association between BMI and all-cause mortality did not change and that more fine-grained risk stratification was achieved for ABSI in each BMI stratum. ABSI is a reliable predictor of diseases such as cardiovascular disease and all-cause mortality and a valid proxy for identifying sarcopenic obesity, independent of BMI by design, and is a useful complement to BMI for risk estimation and risk stratification [22, 43–45].

In this study, we also analyzed the correlations between BMI and WC and ABSI, and in agreement with the results of previous studies [19], there was a strong correlation between BMI and WC and almost no correlation between BMI and ABSI (r < 0.05); in addition, in the association analysis of single indicators with NAFLD risk we found that BMI, WC, and ABSI were all significantly and positively associated with the risk of NAFLD (Table 4). Therefore, the combination of BMI and ABSI may be more appropriate than the combination of BMI and WC. Nevertheless, to fully explore the value of combining BMI and the abdominal obesity index to assess the risk of NAFLD and to circumvent possible statistical artifacts, we still combined BMI with WC and ABSI in 4 separate ways based on previous studies: First, the combination of BMI and WC into four obesity phenotypes, BMI^N^/WC^N^, BMI^O^/WC^N^, BMI^N^/WC^O^, and BMI^O^/WC^O^, based on the overweight cut-offs for WC and BMI in Asian populations recommended by the WHO Expert Committee [27, 28]. Second, constructing optimal proportional combinations O_BMI+WC_ and O_BMI+ABSI_ based on the regression coefficients of BMI, WC, and ABSI in multivariate logistic regression models. Third, multiplied BMI with WC and ABSI directly to obtain BMI*WC and BMI*ABSI. Fourth, a predictor ARI (BMI, ABSI) was calculated using the method developed by Krakauer NY et al. [26]. In the analysis of the association between each obesity phenotype and NAFLD we found that after adjusting for a large number of confounders (Model 3), subjects with normal BMI and WC in both sexes had the lowest risk of NAFLD, both the BMI^O^/WC^N^ phenotype and the BMI^N^/WC^O^ phenotype increased the risk of NAFLD, and subjects with the BMI^O^/WC^O^ phenotype had the highest risk of NAFLD (male: OR 4.19, 95%CI 3.54, 4.96; female: OR 9.78, 95%CI 7.35, 13.02); these results were in accord with previous studies [18]. Obviously, comprehensive consideration of the effects of BMI and WC on NAFLD in the general population could explain the obesity-related NAFLD risk to a greater extent, identifying more populations at risk for developing NAFLD. Additionally, all other combined indicators in this study were significantly and positively correlated with NAFLD, with BMI*ABSI having the strongest correlation among males and BMI*WC having the strongest correlation with NAFLD among females. The results also did not change after treating BMI*ABSI as a categorical variable, but it is worth mentioning that the Q4 category has a much higher risk of NAFLD in females than in males compared with the Q1 category in BMI*ABSI (OR: female 63.97 vs. male 26.15). Consistently, in the obesity phenotypes, female subjects with BMI^O^/WC^O^ phenotype also had a significantly higher risk of NAFLD than males (OR: female 9.78 vs. male 4.19). This suggested that elevated BMI and WC and ABSI may be more important risk factors for NAFLD in females, and females should pay more attention to maintaining normal body weight and body shape to prevent the occurrence of NAFLD.

Our current study also compared the ability of five continuous combined indicators, ARI, O_BMI+WC_, O_BMI+ABSI_, BMI*ABSI, and BMI*WC, to identify NAFLD with BMI and WC and ABSI alone. According to the results of ROC analysis, ARI and O_BMI+ABSI_ had the strongest NAFLD identification ability in both sexes, and although there were only minor differences between the two and O_BMI+WC_, BMI*WC and BMI*ABSI, they were all significantly higher than BMI and WC and ABSI alone. From Table 5 we found that the optimal proportional combination indicators (O_BMI+WC_ and O_BMI+ABSI_) have extremely similar diagnostic efficacy to the multiplicative indicators (BMI*WC and BMI*ABSI) and ARI, which was an interesting result. The optimal proportional combination indicators in this study were calculated after directly obtaining the proportional coefficients based on the ratio of the regression coefficients of BMI and WC or ABSI in model 3, which considered the relative weights of the effects of BMI and WC or ABSI on NAFLD and could fully reflect the effects of BMI combined with WC and ABSI on NAFLD. However, the construction of the calculation formula for the optimal proportional combination was relatively complicated, which may be affected by different adjusted confounding factors and changes in the correlation between BMI, WC, ABSI and NAFLD in different test groups. In contrast, the ARI (BMI, ABSI) was obtained by multiplying the logarithms of ORs of BMI and ABSI by the corresponding BMI and ABSI values ​​of each subject and then summing them together [26] and the multiplicative combination indicators were obtained by directly multiplying BMI with WC and ABSI, which are easy to calculate and had a high diagnostic performance in both sexes. In summary, ARI and multiplicative combination indicators (BMI*WC and BMI*ABSI) may be better obesity indicators for clinical screening of NAFLD. Additionally, the results of the ROC analysis after further grouping the sexes by different age groups showed that all combination indicators of BMI and ABSI or WC significantly improved the ability of a single indicator to identify NAFLD in middle-aged females and young and middle-aged males. It is worth mentioning that the highest diagnostic efficacy for NAFLD was found in the young female population for both combined and single obesity indicators; this may be related to the protective effect of large amounts of estrogen on NAFLD in young females. As we all know, the reproductive status of females is closely related to their health status. Experimental studies have shown that estrogens can exert antioxidant, anti-steatosis, and anti-fibrotic effects in the liver [46, 47]. Whereas, a significant decrease in estrogen levels and an increase in circulating androgen levels in postmenopausal females may lead to disorders of lipid metabolism and the development of metabolic syndrome [48]. In addition, the decline in the body’s metabolic status with aging is also an important risk factor for NAFLD [49]. Thus, younger females have a healthier metabolic profile and fewer confounding factors that affect the risk of NAFLD identified by obesity indicators compared with postmenopausal females and males.

Our findings have important implications for both clinical screening and epidemiological studies of NAFLD. Due to the insidious onset of NAFLD, early-stage patients may be asymptomatic, primary care workers lack attention to NAFLD, and public awareness and treatment rates are very low [50, 51]; moreover, there are no approved treatments for NAFLD, hence the mainstay of prevention and treatment of NAFLD remains healthy lifestyle and weight control [1]. In the current study, ARI and BMI*WC and BMI*ABSI had greater than 80% diagnostic accuracy in all age groups of both sexes and up to 95.22% in young women, and ARI and BMI*WC and BMI*ABSI were easily calculated without additional measurements or equipment requirements. Therefore, we recommend that the ARI and BMI*ABSI and BMI*WC should be incorporated into the clinical screening plan for NAFLD, which will greatly improve the detection rate of NAFLD.

### Advantages and limitations of research

Several advantages of the current study need to be mentioned: (1) The current study demonstrated for the first time in a large sample of the general population that BMI combined with WC and ABSI had a stronger ability to identify NAFLD than a basic anthropometric obesity parameter. (2) After combining BMI with WC and ABSI in various ways, we found that the ARI and multiplicative combination indicators (BMI*WC and BMI*ABSI) may be the more appropriate obesity index for clinical screening of NAFLD. These findings provided an important reference for the application of BMI combined with the abdominal obesity index for NAFLD screening in the general population.

Of course, it is undeniable that the current study has several limitations: (1) The current study was a cross-sectional study, so we can only demonstrate that BMI combined with the abdominal obesity index was stronger than a single indicator for identifying NAFLD in the general population, but whether the predictive performance for NAFLD risk is stronger needs to be validated in a longitudinal cohort study. (2) The diagnosis of NAFLD, the main outcome in this study, was based on ultrasonography, which may have missed some patients with NAFLD [1]. However, in the setting of fewer NAFLD cases, we still demonstrated that BMI combined with the abdominal obesity index had a higher diagnostic performance for NAFLD than a single indicator. (3) Although we systematically adjusted for known NAFLD risk factors, there may be residual confounding due to the limitations of retrospective studies where some NAFLD risk factors could not be measured and obtained.

## Conclusion

Taken together, our findings confirmed that BMI combined with ABSI and WC can better explain obesity-related NAFLD risk than a single indicator in the general population, and identified the ARI and the multiplicative combination indicators BMI*ABSI and BMI*WC as the simplest and very effective combination indicators for diagnosing the risk of NAFLD. These new findings underscored the importance of the combined application of BMI and the abdominal obesity index in clinical practice to assess NAFLD risk in the general population and provided a cost-effective tool for precise preventive screening and treatment monitoring for NAFLD.

## Electronic supplementary material

Below is the link to the electronic supplementary material.

Supplementary Material 1

Supplementary Material 2

## Data Availability

The data used in this study have been uploaded to the “Dryad” database by Professor Okamura et al.

## Abbreviations

BMI: Body mass index
WC: Waist circumference
ALT: Alanine aminotransferase
AST: Aspartate aminotransferase
GGT: Gamma-glutamyl transferase
HDL-C: High-density lipoprotein cholesterol
TC: Total cholesterol
TG: Triglyceride
FPG: Fasting plasma glucose
HbA1c: Hemoglobin A1c
SBP: Systolic blood pressure
DBP: Diastolic blood pressure
NAFLD: Non-alcoholic fatty liver disease
VIF: Variance inflation factor
ROC: Receiver operating characteristic
AUC: Area under the curve
BMI^N^/WC^N^: Normal weight + normal WC
BMI^O^/WC^N^: Overweight/obese + normal WC
BMI^N^/WC^O^: Normal weight + central obesity
BMI^O^/WC^O^: Overweight/obese + central obesity
OR: Odds ratio
CI: confidence interval
ABSI: A body shape index
ARI: Anthropometric risk index
