# Supplementary material for: The value of combining the simple anthropometric obesity parameters, Body Mass Index (BMI) and a Body Shape Index (ABSI), to assess the risk of non-alcoholic fatty liver disease
Source: Lipids Health Dis. 2022 Oct 20;21:104. doi: 10.1186/s12944-022-01717-8 (PMC9585710; doi:10.1186/s12944-022-01717-8)
Supplement: Supplementary file 2 — Supplementary Material 2 [file 12944_2022_1717_MOESM2_ESM.docx]

Supplementary Table 1: Collinearity diagnostics steps.

|  | Variance inflation factor | | | | | | | |
| --- | --- | --- | --- | --- | --- | --- | --- | --- |
|  | Step 1 | Step 2 | Step 3 | Step 4 | Step 5 | Step 6 | Step 7 | Step 8 |
| BMI*WC | 844.2 | 844.2 | 141 | 140.9 | 123 | 13.2 | 1.8 | 1.8 |
| Sex | 136.6 | 136.6 | 109.8 | 21.1 | 3.3 | 3.3 | 3.3 | 3.3 |
| Age | 1.4 | 1.4 | 1.4 | 1.4 | 1.4 | 1.4 | 1.3 | 1.3 |
| Height | 68.8 | 68.8 | 59.7 | 48.1 | 24.8 | 2.8 | 2.4 | 2.4 |
| Weight | 969.3 | 969.3 | 285.3 | 115.5 | 82 | NA | NA | NA |
| BMI | Inf | NA | NA | NA | NA | NA | NA | NA |
| O_BMI+WC_ | Inf | 7260.4 | 2125.9 | NA | NA | NA | NA | NA |
| O_BMI+ABSI_ | 1915.4 | 1915.4 | 1532.1 | 237.2 | NA | NA | NA | NA |
| WC | Inf | 1184.5 | 87.7 | 65.7 | 32.5 | 14.4 | NA | NA |
| BMI*ABSI | 8041 | 8041 | NA | NA | NA | NA | NA | NA |
| Drinking | 1.2 | 1.2 | 1.2 | 1.2 | 1.2 | 1.2 | 1.2 | 1.2 |
| ALT | 4.1 | 4.1 | 4.1 | 4.1 | 4.1 | 4.1 | 4.1 | 4.1 |
| DBP | 5.6 | 5.6 | 5.6 | 5.6 | 5.6 | 5.6 | 5.6 | NA |
| SBP | 5.5 | 5.5 | 5.5 | 5.5 | 5.5 | 5.5 | 5.5 | 1.4 |
| AST | 3.3 | 3.3 | 3.3 | 3.3 | 3.3 | 3.3 | 3.3 | 3.3 |
| Smoking | 1.4 | 1.4 | 1.4 | 1.4 | 1.4 | 1.4 | 1.4 | 1.4 |
| Exercise | 1 | 1 | 1 | 1 | 1 | 1 | 1 | 1 |
| GGT | 1.5 | 1.5 | 1.5 | 1.5 | 1.5 | 1.5 | 1.5 | 1.5 |
| HDL-C | 1.8 | 1.8 | 1.8 | 1.8 | 1.8 | 1.8 | 1.8 | 1.8 |
| HbA1c | 1.2 | 1.2 | 1.2 | 1.2 | 1.2 | 1.2 | 1.2 | 1.2 |
| TC | 1.5 | 1.5 | 1.5 | 1.5 | 1.5 | 1.5 | 1.5 | 1.5 |
| TG | 1.7 | 1.7 | 1.7 | 1.7 | 1.7 | 1.7 | 1.7 | 1.7 |
| FPG | 1.5 | 1.5 | 1.5 | 1.5 | 1.5 | 1.5 | 1.5 | 1.5 |

Note-1: Variance inflation factor = 1/(1-R^2^). Abbreviations as in Table 1.

Note-2: The variables with Variance inflation factor >5 will be regarded as collinear variables and cannot be included in the multiple regression model.

Supplementary Table 2: correlations between the anthropometric measures

|  | BMI | WC | ABSI | Height |
| --- | --- | --- | --- | --- |
| BMI | 1 | 0.8191^**^ | 0.0350^**^ | -0.1021^**^ |
| WC | 0.8796^#^ | 1 | 0.5797^**^ | 0.0927^**^ |
| ABSI | 0.0220^#^ | 0.4575^#^ | 1 | 0.0510^**^ |
| Height | -0.0121^#^ | 0.1710^#^ | 0.0088^#^ | 1 |

Abbreviations as in Table 1.

**Strength of correlations between anthropometric measures in female subjects.

# Strength of correlations between anthropometric measures in male subjects.

Supplementary Table 3: Odds ratios and 95% confidence interval of obesity phenotypes for NAFLD in females and males.

|  | Odds ratios (95% confidence interval) | | | |
| --- | --- | --- | --- | --- |
|  | Crude model | Model 1 | Model 2 | Model 3 |
| Female |  |  |  |  |
| Phenotypes |  |  |  |  |
| BMI^N^/WC^N^ | 1.0 | 1.0 | 1.0 | 1.0 |
| BMI^O^/WC^N^ | 9.98 (6.18, 16.12)^*^ | 8.50 (5.22, 13.85)^*^ | 5.08 (3.03, 8.52)^*^ | 3.67 (2.13, 6.33)^*^ |
| BMI^N^/WC^O^ | 7.08 (5.33, 9.41)^*^ | 6.15 (4.58, 8.26)^*^ | 4.80 (3.53, 6.53)^*^ | 3.77 (2.74, 5.19)^*^ |
| BMI^O^/WC^O^ | 29.09 (22.89, 36.97)^*^ | 27.31 (21.41, 34.84)^*^ | 16.28 (12.54, 21.13)^*^ | 9.78 (7.35, 13.02)^*^ |
| Male |  |  |  |  |
| Phenotypes |  |  |  |  |
| BMI^N^/WC^N^ | 1.0 | 1.0 | 1.0 | 1.0 |
| BMI^O^/WC^N^ | 5.43 (4.33, 6.81)^*^ | 5.11 (4.07, 6.42)^*^ | 4.00 (3.14, 5.09)^*^ | 2.76 (2.12, 3.59)^*^ |
| BMI^N^/WC^O^ | 4.17 (3.49, 4.97)^*^ | 4.59 (3.82, 5.52)^*^ | 3.74 (3.07, 4.54)^*^ | 2.74 (2.22, 3.38)^*^ |
| BMI^O^/WC^O^ | 11.98 (10.42, 13.76)^*^ | 12.51 (10.86, 14.40)^*^ | 8.24 (7.10, 9.58)^*^ | 4.19 (3.54, 4.96)^*^ |

^*^*P*< 0.0001;

Model 1 adjusted for age and height.

Model 2 adjusted for model 1 plus TC, TG, HDL-C, drinking status.

Model 3 adjusted for model 2 plus ALT, AST, GGT, FPG, HbA1c, and SBP.
